# Assessing the Vulnerability of Eco-Environmental Health to Climate Change

**DOI:** 10.3390/ijerph7020546

**Published:** 2010-02-12

**Authors:** Shilu Tong, Peter Mather, Gerry Fitzgerald, David McRae, Ken Verrall, Dylan Walker

**Affiliations:** 1 School of Public Health and Institute of Health and Biomedical Innovation, Queensland University of Technology, Kelvin Grove, Brisbane, Qld. 4059, Australia; E-Mail: gj.fitzgerald@qut.edu.au; 2 School of Natural Resource Science, Queensland University of Technology, Gardens Point, Brisbane, Qld. 4001, Australia; E-Mail: p.mather@qut.edu.au; 3 Queensland Climate Change Centre of Excellence, Department of Environment and Resource Management, Indooroopilly, Brisbane, Qld. 4068, Australia; E-Mail: David.Mcrae@climatechange.qld.gov.au; 4 Environmental and Technical Services, Department of Environment and Resource Management, Indooroopilly, Brisbane, Qld. 4068, Australia; E-Mail: ken.verrall@epa.qld.gov.au; 5 Environmental Health Branch, Queensland Health, Herston, Brisbane, Qld. 4006, Australia; E-Mail: Dylan_walker@health.qld.gov.au

**Keywords:** climate change, eco-environmental health, impact, vulnerability

## Abstract

There is an urgent need to assess the vulnerability of eco-environmental health to climate change. This paper aims to provide an overview of current research, to identify knowledge gaps, and to propose future research needs in this challenging area. Evidence shows that climate change is affecting and will, in the future, have more (mostly adverse) impacts on ecosystems. Ecosystem degradation, particularly the decline of the life support systems, will undoubtedly affect human health and wellbeing. Therefore, it is important to develop a framework to assess the vulnerability of eco-environmental health to climate change, and to identify appropriate adaptation strategies to minimize the impact of climate change.

## 1. Introduction

Over the past decades, there have been escalating global environmental changes driven largely by rapid industrialization, exponential population growth, over-consumption of natural resources and associated challenges of waste disposal, and the indiscriminate and inappropriate uses of technology [1–3]. Climate change is, arguably, the most significant environmental challenge facing human society this century.

There is increasing recognition that climate change is affecting and will, in the future, have more (mostly adverse) impacts on ecological systems. For instance, climate change is already affecting living systems. Global meta-analyses documented significant range shifts among 279 different species, averaging 6.1 km per decade towards the poles, and significant mean advancement of spring events by 2.3 days per decade [4]. Significant changes in physical and biological systems are occurring on all continents and in most oceans. Most of these changes are corresponding with warming global and regional temperatures. These changes imply that anthropogenic climate change is having a significant impact on physical and biological systems globally [5].

An assessment of the impact of climate change on eco-environmental health becomes a critical and urgent issue for the scientific community and governments at all levels. Therefore, it is important to examine the vulnerability of eco-environmental health to climate change.

## 2. Definition and Trend of Eco-Environmental Health

Although a variety of ways have been offered to define environmental health in the literature [6–8], the fundamental concept is that *“environmental health addresses all the physical, chemical, and biological factors external to a person, and all the related factors impacting behaviours. It encompasses the assessment and control of those environmental factors that can potentially affect health. It is targeted towards preventing disease and creating health-supportive environments. This definition excludes behaviour not related to environment, as well as behaviour related to the social and cultural environment, and genetics”* [9].

In this context, eco-environmental health refers to ecosystem functions and services (land and water resources, agriculture, biodiversity), and interactions of organisms with their environment and with each other [10,11]. There are strong connections between ecosystem health and human health. Ecosystems are complex and fragile, and are susceptible to human activities. Rapid urbanisation, population growth and intensive consumption can not only jeopardise the health of humans but also the health of the ecosystems that we live on.

According to the World Health Organization (WHO), *“ecosystems are the planet’s life-support systems - for the human species and for all other forms of life. The needs of human biology for food, water, clean air, shelter and relative climatic constancy are basic and unalterable. Ecosystem services are indispensable to the wellbeing of all people, everywhere in the world. The causal links between environmental change and human health are complex because they are often indirect, displaced in space and time, and dependent on a number of modifying forces”* [12].

It is well known that global climate change can directly impact on ecosystem functions and services, and accruing evidence indicates significant ecological effects of climate change [13,14]. Global atmospheric concentrations of greenhouse gases such as carbon dioxide, methane and nitrous oxide have increased markedly as a result of human activities since 1750 [15,16]. Global increases in carbon dioxide concentration is due primarily to fossil fuel use and land-use change, while those of methane and nitrous oxide are primarily due to agricultural inputs [15,17].

The Fourth Assessment Report (AR4) of the Intergovernmental Panel on Climate Change projected an increase of 1.1 to 6.4 ºC in global average surface temperature by 2100, with a greater temperature rise at higher latitudes and over land [15]. Change in global temperatures, in turn, has affected thermal expansion of the oceans, as well as increased melting of glaciers, ice sheets and ice caps and these together have ultimately contributed to global sea level rise over the past century [18]. According to the AR4, however, anthropogenic climate change will continue even if carbon dioxide emissions are reduced immediately, as short-term (e.g., by 2030) climate projections are relatively insensitive to differences in future emissions of greenhouse gases. Relatively small changes in mean climate can result in larger changes in extremes (e.g., heatwaves, floods, cyclones and bushfires) [15].

Climate change can exacerbate significant influences on ecosystems and alter services (beneficial resources and processes) they provide [11,19]. While organisms have adapted to their regional climate over time, climate change alters the resources and processes that they provide to society, and potentially can act as a factor that alters ecosystems, particularly when the change is occurring at an unprecedented fast rate compared with the past. For example, humans depend on ecosystems for the natural, cultural, spiritual, and recreational resources they provide. Global warming can affect biological and ecological components of the ecosystem and can create new environmental conditions for humans and other organisms by changing and disturbing ecosystem dynamics [4,20].

Clearly, the ecosystem will not only be affected by increased temperatures but also by altered rainfall patterns. While global average annual rainfall is projected to increase with climate change, many mid latitude and lower latitude land regions are likely to become drier [15]. Extreme precipitation events (e.g., flooding) could become more severe and frequent in some global regions and climate variability is expected to increase in a warmer world.

There is ample evidence that anthropogenic climate change has already caused a discernible impact on physical and biological systems globally. Most of these changes are in the direction expected with warming global surface temperatures. These changes in natural systems, since at least 1970, are occurring in regions of observed temperature increases, and temperature increases at continental scales cannot be explained by natural climate variation alone [5]. Additionally, extreme events, including hurricanes, floods, storms and severe droughts, can cause major ecosystem disturbance that may impose huge burdens in economic terms and cause adverse effects on human health and well-being [21,22].

## 3. Human Health Vulnerability

Most of the warming observed over the last six decades can be attributed to human activities [15]. Like other species, humans are vulnerable to climate change [2,19,23,24]. Climate change is already affecting, and will increasingly have profound effects on human health and well-being. Responses to some recent extreme climate events have revealed higher levels of vulnerability across the globe, producing significant loss of life and property damage in both developing and developed countries. The large and unexpected health impacts due to a heat wave of unprecedented magnitude in 2003 in Europe provide one such example [25]. Therefore, there is an urgent need for societies to take both pre-emptive and adaptive actions to protect human populations from adverse health consequences of climate change.

Climate change can result in a wide range of potential, mostly adverse, effects on human health. Effects can be classified by whether they occur directly—in relation to physiological effects of weather extremes (e.g., heat, cold, flooding and bushfires), or indirectly such as changes in disease transmission dynamics [24], ecological disruption [2] and/or malnutrition [19].

## 4. Vulnerability to Weather Extremes

### Hot and cold weather

Human health vulnerability to climate change and other eco-environmental disturbances has attracted increasing research attention. For example, as climate change continues, higher average ambient air temperatures are likely to induce more vigorous cycles of evaporation and precipitation. Indeed, a trend of increasing climate variability and extreme precipitation events has been observed over the past century, and recent models strongly correlate this trend with anthropogenic production of greenhouse gases [26,27]. High temperatures cause well known clinical syndromes including heat stroke, heat exhaustion, heat syncope and heat cramps [28–30]. Many studies have reported that human exposure to both extreme hot and cold weather is associated with increased morbidity and mortality [31–34], especially from cardiovascular and respiratory disease in temperate countries [30,35]. The impact of extreme weather events may vary, however, depending on the age structure, socio-demographic factors, high-risk behaviours, health status, and environmental conditions of the people affected [36,37].

At highest risk are the populations that are most exposed (e.g., poor elderly without air conditioning) and those that have the fewest technical and social resources [38]. The health impacts of extreme weather events include physical injury; poorer nutritional status, especially in children; increases in respiratory and diarrhoeal diseases due to overcrowding of flood survivors and limited access to potable water; increased risk of water-related diseases due to disruption of water supply or sewage systems; and release of dangerous chemicals from storage sites and waste disposal sites into flood waters [39].

### Floods

Climate change is also predicted to cause more flooding in some regions due to more frequent, heavy rainfall events [40]. Coastal communities are especially vulnerable to coastal surges exacerbated by a combination of rising sea levels and more intense storms [4].

Floods have direct immediate effects on mortality as well as longer term effects. For example, populations that have experienced flooding may suffer from sustained increases in common mental disorders [41].

Depending on catchment size, topography, and climate, floods can cause immediate deaths and injuries from drowning and from collapsing infrastructure or floating debris [42]. In addition, floods can influence the spread of diseases (from disruption of water purification and sewage systems), food shortages, exposure to airborne allergens and toxic chemical releases. The health impacts of floods may be greater when industrial or agricultural land adjoining residential land is affected. Mobilization of chemicals already in the environment or from storage may also occur during floods [43]. Increases in diarrhoeal and respiratory disease, post floods, are often reported, in both developed and developing countries [41,44]. In general, the health risk from floods is influenced by both the characteristics of the flood (e.g., its scale and duration, the suddenness of onset, the velocity and depth of water, the lack of warning) and of the population that it affects. Floods with the largest mortality impacts have occurred where infrastructure is poor and the population at risk has limited economic resources [41].

### Droughts

Droughts can have wide ranging effects on health including those on nutrition, infectious diseases, and on forest fires causing air pollution, particularly in developing countries. Drought-related bushfires can cause direct injury and have the potential to affect air quality by producing smoke and other airborne particulates. Fire smoke carries a large amount of fine particles that can increase hospital admissions from asthma and other respiratory diseases [45,46]. Droughts can also produce diverse impacts on human health by changing patterns of nutrition, infectious diseases, and suicide and mental disorder rates among farmers [47,48].

There are other weather extremes such as cyclones, storms and hurricanes which also have significant impacts on community health and well-beings. The list of weather extremes here is not exhaustive.

## 5. Vulnerability to Other Impacts

Climate change will have increasing impacts on ecosystem functions and services that will undoubtedly affect human health and wellbeing. Many of these impacts are already observed in different regions of the world [1,2]. Vulnerability of eco-environmental health to climate change, therefore, has received increasing research attention. The following are examples to illustrate this issue.

### Food security

One of the most profound impacts of climate change is likely to be on agricultural systems and food production [49]. Changes in mean daily temperature and rainfall can cause severe plant disease epidemics [50,51] that may threaten food security [21,52] and can disturb ecosystems and affect extinction probabilities of many species [5,53].

For example, increases in mean temperature and a decline in precipitation rates are likely to reduce yields of corn, wheat, rice, and other stable food crops [54,55]. These changes, in conjunction with changes in land use that result from urbanization and agriculture, are likely to impact substantially on global food production and food security [56]. The overall impact of climate change on food security will depend on the socio-economic status of each affected country [57] and the extent of climate change in different regions [54,55]. There is little empirical information available in the literature, however, on the inter-relationship between climate change, food security and human health.

### Freshwater

Water is essential to human health and survival. The amount of freshwater on the Earth is finite but its distribution varies substantially with region [58,59]. While most of the world’s drinking water supplies are extracted from rivers and groundwater systems, both are sensitive to long-term changes in precipitation rates, temperature, and melting of snowcaps and glaciers [60].

In a rapidly urbanising world, water shortage and water pollution are serious problems, particularly in developing countries. Nearly 1.5 billion people worldwide lack safe drinking water, and at least 5 million deaths per year can be attributed directly to waterborne diseases [61]. Inadequate provision for solid waste collection frequently results in faecal contamination of water bodies that, coupled with high population densities—an inherent problem in most cities, presents a substantial risk for epidemics to spread rapidly.

Overall, when the flow of water is too great in a drainage system, as a result of heavy rainfall or snowmelt, waterborne pathogens are spread in contaminated drinking water, exposure to contaminated water while swimming or other activities, or secondarily via food contaminated with bad water [62]. For drinking water to be a source of illness, the water must first be contaminated, escape treatment, or treatment must fail. Human sewage, leaking septic systems, manure runoff from agricultural lands, and wild animal wastes may all contaminate surface water later used for drinking water. Groundwater may become contaminated by overuse of agricultural chemicals and fertilizer, surface contamination of wells, subsurface inflows, septic fields that are poorly designed, or leaking dumps (chemical contamination). Drinking water can also become contaminated during or after water treatment [63].

The main impacts of climate change on human populations and the environment in general are likely to occur through water [19,63], but the causal links between climate change, quantity and quality of freshwater resources and human health remain to be determined.

### Infectious diseases

Although many factors (e.g., social, economic, biological and ecological factors) can influence the transmission of infectious diseases, climate change is an emerging risk factor for infectious diseases because it has the potential to affect the reproduction and survival of both infectious agents (e.g., protozoa, bacteria, fungi and viruses) and their associated vector organisms (e.g., mosquitoes, ticks and sandflies), and hence their relative ability to infect humans [64–66]. In the meantime, climate change will also influence human behaviour (e.g., amount of outdoor activity) that may increase exposure of individuals to infectious vectors [1,19].

Malaria, as an important human pathogen, for example, is well-known to be sensitive to climate effects. Half of the world's population (about 3.3 billion) is at risk of malaria, and an estimated 247 million cases led to nearly 881,000 deaths in 2006 [67]. Wet and humid environments provide more breeding sites for malarial-vector mosquito species and can also prolong their life span [68,69]. Temperature also governs the rate at which mosquitoes complete development to adults, determines how frequently they blood-feed (and, therefore, acquire parasites), affects adult mosquito survival rates, and determines the incubation time of the parasite in the mosquito [69]. Therefore, malaria epidemics are generally associated with seasonally warm and wet conditions [39,70].

The present and future impact of climate change on malaria and other infectious diseases, however, is a highly debated issue [71–74]. The IPCC has projected an increase and/or change in the geographical range of malaria and some other infectious diseases “with high confidence” [15]. Although this is a very complex issue, evidence is emerging that significant warming in the East African highlands may have contributed to malaria resurgence in these regions [75,76].

Other climate-sensitive vector-borne diseases include dengue fever, schistosomiasis, encephalitis (e.g., Japanese encephalitis), chikungunya fever and Ross River virus infection [65,77–80]. As emergence and resurgence of infectious diseases are driven by many factors, including both climate and non-climate factors such as increasing drug resistance of pathogens, deteriorating public health infrastructure, and decreasing funding for vector control programs in some countries, it is often difficult to disentangle the role played by climate change from the effects of other factors. Thus, there remains a need for quantitative studies that consider these major risk factors, to assess their relative importance in disease transmission in order to provide a scientific basis for future disease control and prevention policy-making.

Globally, waterborne diseases and sanitation-related infections have been identified as major contributors to the world-wide burden of human disease and mortality [81]. Many different viral, bacterial and parasitic diseases are associated with waterborne transmission [82]. Recent studies show that climate can impact on the epidemiology of waterborne disease in three ways; after heavy rainfall events and flooding and following high ambient temperatures [83,84]. For example, heavy rainfall can lead to storm water runoff into surface water sources, which will significantly increase the levels of indicator bacterials as well as potential pathogens [81]. Flooding often follows heavy rainfall, or results from a rise in sea level, both of which can result in flows of contaminated water into groundwater resources [83]. Outbreaks of Leptospirosis in Rio de Janeiro [85] have often followed significant flooding events. Waterborne diseases associate with blooms of various planktonic species (including blue-green algae) that occur following increased temperatures can also be harmful to human health [86]. Studies show that most blooms occur during the summer months and are associated with relatively high water temperatures [87].

There is also increasing research interest in the assessment of climate impacts on spread of food-borne diseases [88,89]. Overall, changes in climate that can affect potential transmission of infectious diseases include temperature, humidity, altered rainfall, raised soil moisture and rising sea levels. Additional factors including socio-economic conditions, disease-control programs, human immunity levels and local land-use changes can also influence transmission cycles of infectious diseases [90–92]. Thus taken together, projected increases in ambient temperatures and changing patterns of rainfall worldwide linked to climate change, have the potential to increase the transmission of infectious diseases [33,93].

### Climate change-related migration

Migration has always been an important mechanism used to deal with climate stress, and archaeological evidence suggests that changes in human settlement patterns were often a response to changes in climate over history [1,94].

Climate induced human migration or population dislocation can cause diverse risks to human health and wellbeing, especially in small island areas with high population densities, a fragile resource base, and/or low economic resilience [95].

There are also other impacts such as desertification and salinity associated with climate change. These environmental alteration/changes can not only disturb the health of humans but also the health of the ecosystems that support us.

## 6. Measures of Eco-Environmental Health Vulnerability

In the context of environmental health research, “vulnerability” was first used by Witt and Reiff to isolate environmental health conditions and potential points of contamination responsible for a cholera outbreak in Latin America and the Caribbean [96].

Cutter indicated: “vulnerability is the likelihood that an individual or group will be exposed to and adversely affected by a hazard”, which defined vulnerability as risk of exposure to hazards and a capability for social response (referred to ‘coping’ or ‘adaptive capacity’ below) [97].

A more comprehensive definition of vulnerability was provided by IPCC [15] as: *the degree to which a system is susceptible to, or unable to cope with, adverse effects of climate change, including climate variability and extremes. Vulnerability is a function of the character, magnitude, and rate of climate variation to which a system is exposed, its sensitivity, and its adaptive capacity*. Overall, a broad definition of vulnerability is “the likelihood to be harmed,” [19] and can be applied to most areas of research. Therefore, vulnerability is a function of exposure and sensitivity (impacts) on the one hand, and the ability of affected systems to adapt to change (adaptive capacity) on the other.

A few approaches have been trialled for assessing vulnerability of eco-environmental health to climate variability and change [11,19]. For example, the Millennium Ecosystem Assessment project attempted to address the inter-relationships between climate change, ecosystem degradation and human health [11]. Frameworks were proposed for assessing the potential impacts of climate variability and change on physical and biological environment [5] or on human health [95], but few data are available on the assessment of inter-relationships between climate change, ecosystem degradation and human health.

Quantitative assessments are vital for understanding potential implications of climate change and associated impacts. One approach is to use analogues from past climate change events. A large literature base is available on changes to the physical environment following previous climate change events, including, for example, effects on land cover [98,99] and ecosystems by large [100,101]. The use of expert judgement to assess implications of defined climate change is another quantitative approach that has been adopted [102]. This approach is the basis for IPCC assessment of the impacts of climate change [19].

Various methods have been developed for quantitative estimation of health impacts of future climate change [65,66,70,71,103]. All rely on the study of climate effects on health in the past, in the present (e.g., climate as a risk factor of current disease distribution), or process-based computer modelling. However, these studies encountered enormous difficulties in taking into account confounding factors as data on many of these factors are scarcely available.

The assessment of health consequences of eco-environmental change is challenging. In traditional environmental epidemiology, only a proportion of the population is ever exposed to any pollutant of concern. Quantifying this exposure is therefore an important step in the risk assessment process [104]. In climate change assessment, for example, the whole population is assumed to be exposed to changes in climate, although the degree of change may vary spatially and temporally [15]. Recently, the WHO proposed six key themes in the assessment of health impacts of climate change, including [105]:

1. Research on the health effects of climate change must be placed more firmly within the overall context of improving global health, and health equity, rather than being considered to stand alone.
2. There is a need to improve policy-relevant risk assessment, building a stronger bridge between evaluation of the existing health risks from short-term to medium-term climate variability and the effects of gradual climate change, in the context of other drivers such as socioeconomic development and urbanisation.
3. A comprehensive evaluation of the effectiveness, and cost-effectiveness, of protective measures is required.
4. Applied research is needed to maximise the health benefits of decisions taken beyond the health sector.
5. Improved research on surveillance and other decision-support tools is necessary to enhance operational effectiveness.
  Economic assessments of the costs and benefits of mitigation and adaptation decisions are crucially important.

All these themes are fundamentally important and require substantial investment. In order to fill these research gaps, it is necessary to prioritise research needs, build interdisciplinary research capacity and provide more funding in this emerging and challenging area.

### Adaptive capacity

Adaptive capacity refers to the potential, capability, or ability of a system to adapt to the impacts of climate change [19]. Adaptation can be anticipatory (prepare for hazards and opportunities in advance) or responsive (respond or cope with the effects) and can encompass both spontaneous responses to climate variability and change by affected individuals and planned responses by governments or other institutions [106]. Across human history, societies have adapted to natural climate variability by altering settlement and agricultural practices and other activities. Capacity is the ability of a society to adapt to changing climatic conditions, or other new circumstances, that will depend on the level of wealth, education, institutional strength, access to technology and also to political will [107,108]. Adaptive capacity will, therefore, greatly influence the vulnerability of communities and regions to climate change effects and hazards [109–112]. Thus, the significance of climate variation or change will depend on the change itself and the characteristics of the regions that experience the change [19]. This complex mix of conditions will determine the capacity of any system to adapt to future climate change impacts.

It is widely understood that poverty is directly related to vulnerability and wealthy nations are generally better prepared to bear any costs of adaptation to climate change impacts and risks compared with poorer nations [1,33]. Adaptation to climate change and risks takes place in a dynamic social, economic, technological, biophysical, and political context that will vary over time. It is also known to vary, depending on relative availability and access of a region to technological advances, social infrastructure and knowledge [113–115].

Enhancing adaptive capacity is necessary to reduce vulnerability, particularly in the most vulnerable regions, nations, or socioeconomic groups [108–110]. Activities required for enhancement of adaptive capacity are essentially equivalent to those that promote sustainable development and equity. Clearly, more research is urgently needed in this challenging area. Recently, the National Climate Change Adaptation Facility of Australia [116] developed the criteria for setting research priorities (Table 1). It is anticipated that these criteria will also assist with the assessment of potential impacts of climate change on eco-environmental health.

### Knowledge gaps

The knowledge gaps to date in the field of climate change and eco-environmental health include:

1. How to best define the concept of eco-environmental health?
2. What strategies are needed for developing the conceptual framework and practical processes to assess the impact of climate change on eco-environmental health?
3. Which models can be used to quantify the effects of climate change on eco-environmental health?
4. What are key confounding factors in the examination of the causal relationship between climate change and eco-environmental health?
5. What skills and techniques are urgently required for capacity building in this field?

Evidently, more research is clearly needed to address these issues. Therefore, concerted efforts and more investment are required form relevant government agencies and funding bodies to support research on these topics.

## 7. Conclusion

Anthropogenic climate change is unequivocal. Global warming resulted from human activities in this century will be much greater than that observed in the last century if the condition of ‘business as usual’ continues. There are numerous potential impacts of climate change on eco-environmental health as described above. While some beneficial effects are expected (e.g., short-term increase of soy products in some regions), the overall primary impact of climate change on population health is likely to be adverse. Vulnerability and adaptive capacity are major issues to be addressed, and it is therefore important to examine adaptation strategies as a key element when developing or formulating policies to tackle climate change impacts.

In order to assess the vulnerability of eco-environmental health, there is a need to develop a knowledge base and interdisciplinary expertise in this field. More regional and local research is urgently needed, which may serve as a base for decision-making for eco-environmental recovering and rebuilding. It is vitally important for scientists to work with decision-makers and stakeholders in order to tackle this significant challenge at global, national and local levels. It is time to mainstream eco-environmental health risks and their prevention in relation to global climate change on the scientific research and policy agenda.

## Figures and Tables

**Table 1.** Criteria for setting research priorities.

*Item*	*Content*
**Severity of potential impact/degree of potential benefit**	What is the severity of the potential impact to be addressed or benefit to be gained by the research? Potentially irreversible impacts and those that have a greater severity (in social, economic or environmental terms) will be awarded higher priority.
**Immediacy of required intervention / response**	Research will be prioritised according to the timeliness of the response needed. How immediate is the intervention or response needed to address the potential impact or create the benefit? Research that must begin now in order to inform timely responses will receive a higher priority than research that could be conducted at a later date and still enable a timely response.
**Need to change intervention / practicality of intervention**	Is there a need to change the intervention used currently to address the potential impact being considered. If yes, what are the alternatives and how practical are these alternate interventions? Research that will contribute to practicable interventions or responses will be prioritised. Does research into the potential impact of the intervention being considered contribute to the knowledge base required to support decisions about these interventions?
**Potential for co-benefit**	Will the research being considered produce any benefits beyond informing climate adaptation strategies?
**Potential to address multiple, including cross-sectoral, issues**	Will the research being considered address more than one issue, including cross-sectoral issues?
**Equity considerations**	Who will benefit from any adaptation strategy?

Source: Adapted from Reference [116].
